# Polymer–Ceramic Composite Membranes for Water Removal in Membrane Reactors

**DOI:** 10.3390/membranes11070472

**Published:** 2021-06-26

**Authors:** Ester Juarez, Javier Lasobras, Jaime Soler, Javier Herguido, Miguel Menéndez

**Affiliations:** Catalysis, Molecular Separations and Reactor Engineering Group (CREG), Aragon Institute for Engineering Research (I3A), University of Zaragoza, 50009 Zaragoza, Spain; juarecisima@gmail.com (E.J.); jlasobra@unizar.es (J.L.); jsoler@unizar.es (J.S.); jhergui@unizar.es (J.H.)

**Keywords:** CCUS, renewable methanol, membrane reactor, water removal membrane, polymer ceramic membrane

## Abstract

Methanol can be obtained through CO_2_ hydrogenation in a membrane reactor with higher yield or lower pressure than in a conventional packed bed reactor. In this study, we explore a new kind of membrane with the potential suitability for such membrane reactors. Silicone–ceramic composite membranes are synthetized and characterized for their capability to selectively remove water from a mixture containing hydrogen, CO_2_, and water at temperatures typical for methanol synthesis. We show that this membrane can achieve selective permeation of water under such harsh conditions, and thus is an alternative candidate for use in membrane reactors for processes where water is one of the products and the yield is limited by thermodynamic equilibrium.

## 1. Introduction

Production of methanol from CO_2_ and renewable hydrogen could be the solution for two of the main problems facing humankind—global warming from greenhouse effects and the depletion of fossil fuels. This idea was proposed by the Olah [1,2,3] and is now being incorporated in several demonstration plants, both in operation [4] and in construction [5,6]. A recent review of the concept of methanol economy was presented by Araya et al. [7]. The interest in the development of this technology is evident from the many researchers that have worked on it, as may be seen in several reviews [8,9,10]. Methanol may be used directly as a fuel in internal combustion vehicles or can be transformed to dimethyl-ether (DME); which can also be used as fuel; transformed to gasoline by the well-known methanol-to-gasoline process (MTG); or transformed to olefins (methanol to olefins (MTO) process).

The two main difficulties for the production of renewable methanol as a substitute for fossil fuels are: (a) the high price of renewable hydrogen (i.e., H_2_ produced from renewable energies, such as solar or eolic) compared with hydrogen from non-renewable sources; (b) the high operation pressure, which favors the use of very large-scale plants and is hardly compatible with the use of local and renewable energy sources. The first problem will probably be alleviated in future scenario when CO_2_ use is supported by society, fossil fuels prices have increased, and the cost of renewable hydrogen has decreased. A solution for the second problem may be the use of a membrane reactor. The use of high operation pressures, such as in industrial operations, derives from the need to increase the methanol yield at the reactor exit, which is limited by the thermodynamic equilibrium and is favored by high pressure. The yield to methanol can also be increased by removing one or more reaction products from the reaction environment, according to the Le Châtelier principle. In this way, a higher yield for a given pressure or a lower operation pressure with the same yield can be achieved. In general, removal of a reaction product increases the reaction rate for any reversible reaction. This principle is widely used for process intensification with membrane reactors [11]. Struiss et al. [12,13], who showed both experimentally and by modeling that membrane reactors may allow better yield than conventional reactors, proposed the use of a Nafion membrane; however, the maximum operating temperature of this Nafion membrane was around 200 °C, which is lower than the usual reaction temperature. As such, the reaction kinetics were too slow and the spatial time needed was too large compared with the industrial needs. 

One way to avoid the temperature limitations of Nafion is through the use of zeolite membranes. Menéndez et al. [14] initially proposed the use of a zeolite membrane reactor for methanol synthesis. Gallucci et al. [15] showed experimentally that a membrane reactor using a zeolite A membrane provided higher conversion than a conventional packed bed reactor. Several articles have explored the potential advantages of membrane reactors using mathematical models [16,17]. The effects of the operating conditions on the permeation and selectivity of several zeolite membranes have been studied [18,19], showing that zeolite A provided the best performance. A recent paper [20] showed excellent performance for zeolite A membranes obtained from zeolite nanoparticles. They showed that this membrane can be used in a membrane reactor, providing much better CO_2_ conversion and space time yields than those reported in the literature with conventional reactors. 

An alternative to using a zeolite membrane could be the use of composite (polymer–ceramic) membranes using materials other than Nafion. Polymer–ceramic composite membranes have been widely studied as a way to improve the properties of polymer membranes, endowing them with the high mechanical and thermal properties of ceramic membranes [21]. The polymer properties often improve because of the confinement effect. Otherwise, they could be an alternative to the complex procedure for zeolite membrane synthesis. Chen and Yuan [22] showed the use of a silicone–ceramic membrane for a membrane reactor, although the details of the preparation procedure were not clear. 

The objective of this study is the preparation of a polymer–ceramic composite membrane and the measurement of its permeation properties under conditions simulating the reaction atmosphere during the CO_2_ hydrogenation reaction to methanol. In particular, a high-temperature silicone is deposited on the surface of an alumina membrane and the permeance and permeoselectivity of the different gaseous species are measured. A membrane able to provide selective water permeation at sufficiently high temperatures could be useful, not only for the use of membrane reactors for methanol production, but also in several processes for which the removal of water may improve the yield [23,24,25].

## 2. Experimental System

### 2.1. Membrane Preparation

Ceramic membranes made by Inocermic GmbH (Hermsdorf, Germany), with a thin layer with 200 nm pores, were used as supports. They had an external diameter of 1 cm and internal diameter of 7 mm. The membranes were enameled at both ends with a glaze before the silicone was deposited, leaving a permeable section measuring 5 cm in length. A silicone layer was deposited on the inside of the tube. Two methods were tested, as listed below:

Single silicone layer: A thin layer of RTV 801 silicone manufactured by Mardisur (Málaga, Spain) was deposited on the inside of the tube using an evaporative coating from a hexane solution. Typically, 4 mL of solution with 7.5 wt% of silicone was introduced inside the support, together with 0.1 mL of polymerization catalyst (dibutyl–tin dilaurate, Mardisur, Málaga, Spain). The solution was allowed to slowly evaporate while turning the ceramic tube up and down every 5 min. 

Double silicone layer: The above procedure was repeated twice, using a more diluted (5 wt%) silicone solution. 

It is worth mentioning here that Nafion^®^-supported membranes were also prepared but they decomposed when the temperature approached 200 °C. Other procedures were also tested for the silicone membrane deposition but the resulting membranes had more defects, so they will not be discussed here. 

### 2.2. Membrane Characterization

Membranes were characterized using SEM (JEOL JSM 6400, Tokyo, Japan) after encapsulating them in a resin to avoid detachment of the polymer membrane from the ceramic support. 

Nitrogen permeance was measured at room temperature, then permeance was fitted to the following equation, which assumes that it is the sum of convective and Knudsen contributions:

(1) $$J=J_{l}+J_{Kn}=\frac{\varepsilon r^{2}}{8L\tau \mu RT}P_{m}+\frac{4}{3}\sqrt{\frac{2}{\pi MRT}}\;\frac{\varepsilon r}{L\tau }=A\;P_{m}+B$$

where *J* is the permeance, *J_l_* is the convective contribution to permeance, *J_Kn_* is the Knudsen contribution, *ε* is the membrane porosity, *r* is the pore radius, *P_m_* is the mean pressure, *L* is the membrane thickness, *τ* is the tortuosity, *μ* is the viscosity, *R* is the gas constant, *T* is the temperature, and *M* is the molecular weight. From the slope (A) and the intercept (B), an estimate of the mean pore size was calculated as:

(2) $$r=\frac{A}{B}\;\frac{32\mu RT}{3}\sqrt{\frac{2}{\pi MRT}}$$

This procedure gives an evaluation of the existing defects, since the nitrogen permeation through the dense silicone is expected to be negligible. This approach may be used to estimate the pore size when the contributions from convective and Knudsen flows are similar. 

**Figure 1:**
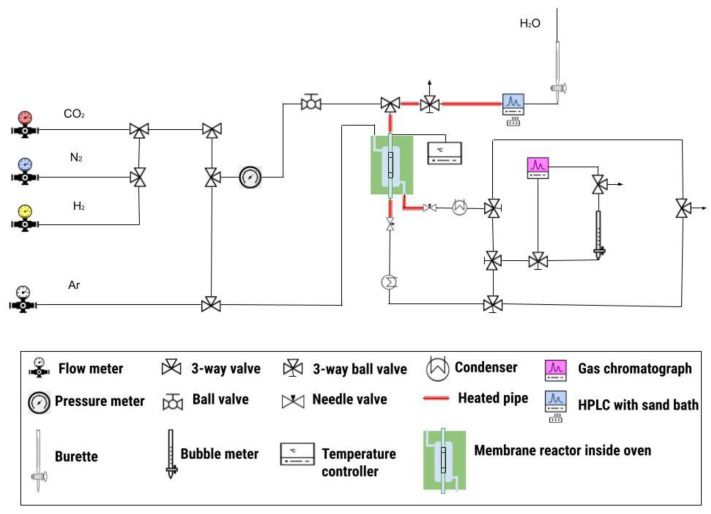
Scheme of the experimental system employed for permeance measurements.

Finally, the selected membranes were inserted into a stainless steel module and sealed with graphite o-rings. A stream containing a mixture of H_2_ (75 cm^3^/min), CO_2_ (25 cm^3^/min), N_2_ (25 cm^3^/min), and water was fed to the internal side, while a stream of Ar (100 cm^3^/min) was fed to the external side, acting as a sweep gas. Water was fed with an HPLC pump ((LC-10AT form Shimadzu, Kyoto, Japan) to an evaporator together with a N_2_ flow to ensure a smooth flow, then mixed with the stream of H_2_ and CO_2_. Both streams were fed at one end of the tube and exited at the other end (parallel flow). The temperature was varied between 180 and 220 °C. The flow of water was varied between 6.9 and 27.4 cm^3^/min (with all volumetric flowrates corresponding to the gas volume measured at STP conditions). The amounts of water in the retentate and permeate streams were measured by condensing and weighting the volumes, and the non-condensable gases were analyzed by gas chromatography. Experiments were performed under atmospheric pressure. Figure 1 shows a scheme of the experimental system.

The permeance of a given compound was calculated as the flux of this gas divided by the logarithmic mean of the difference between its partial pressures in the retentate and the permeate (i.e., the mean of the driving force at the two ends of the membrane). The permeance error was estimated to be approximately 1%.

Selectivity was calculated as the ratio of the permeances of two compounds. 

## 3. Experimental Results

### 3.1. SEM Characterization

**Figure 2:**
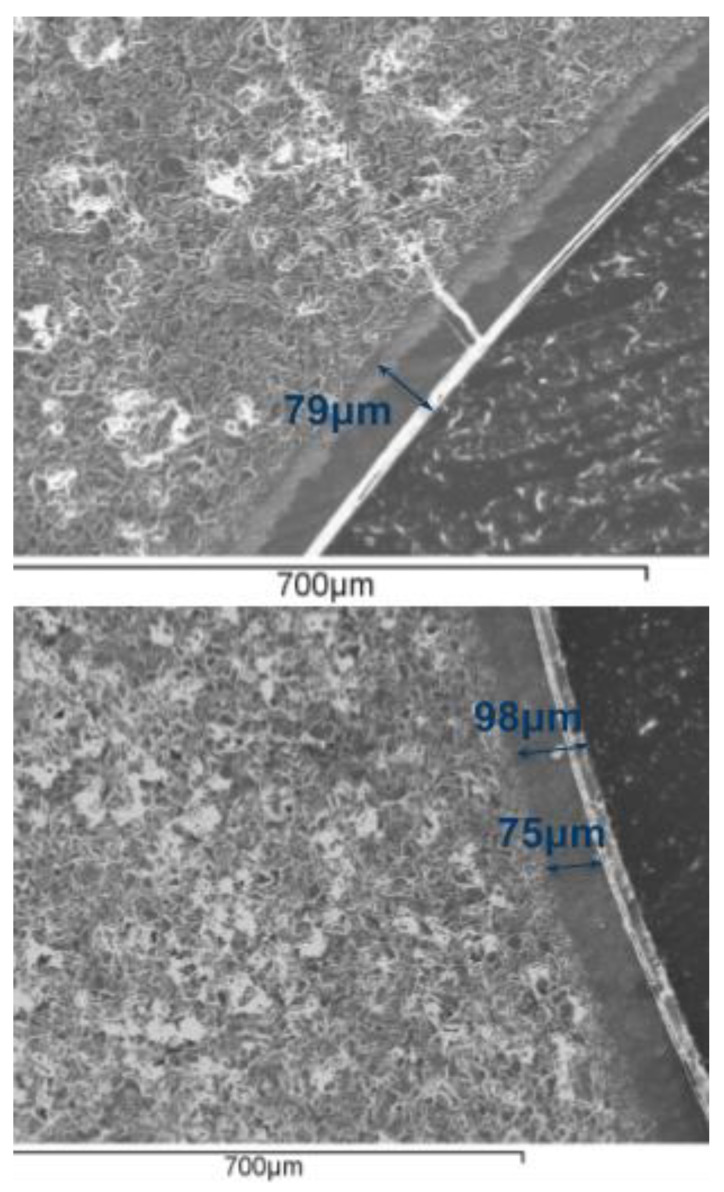
Scanning Electron Microscopy (SEM) image of silicone–ceramic membranes: (**top**) single-layer membrane; (**bottom**) double-layer membrane.

SEM images (Figure 2) showed continuous membranes with thicknesses of around 75 µm deposited on the ceramic support for the single-layer membrane and around 100 µm for the double-layer membrane. In this case, the membrane was slightly different from that used for the permeation test—the solutions used for the preparation of the double-layer membrane had silicone concentrations of 7.5 and 2.5 wt%. The thickness observed using SEM was in agreement with the weight increase observed after membrane preparation (0.01 g/cm^2^ in first case and 0.012 g/cm^2^ in the second). In fact, the layer thickness estimated from the weight increase (with a polymer density close to 1 g/cm^3^) was slightly greater than that measured by SEM, suggesting that some silicone had penetrated into the ceramic pores. In both cases, a continuous polymer layer was deposited on the ceramic support. 

### 3.2. Nitrogen Permeation at Room Temperature

**Figure 3:**
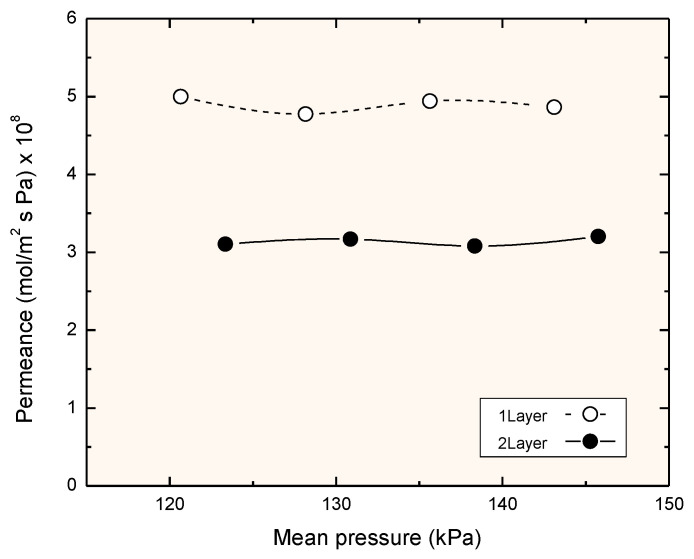
Nitrogen permeation at room temperature.

Nitrogen permeance was measured at room temperature for the two membranes as a function of the pressure. The results are shown in Figure 3 and the fit gives the following equations:

J = 7.6·10^−14^ P_m_ + 3.77·10^−8^ (mol/m^2^ s Pa) for the single layer;

J = 1.96·10^−14^ P_m_ + 2.88·10^−8^ (mol/m^2^ s Pa) for the double layer.

A comparison of both terms showed that the single layer had higher permeance and higher contribution of convective flow, suggesting the existence of more defects. The pore diameters estimated with Equation (2) were 6 nm for the single-layer membrane and 2 nm for the double-layer membrane. Although the large Knudsen contribution probably caused a high relative error in the calculation of the pore size, the difference between both membranes was quite evident.

### 3.3. Permeation Measurements at Reaction Temperature

Table 1 and Table 2 show the permeance levels for each gas calculated from the experimental results with different temperatures and water partial pressures for the two kinds of membranes. 

## 4. Discussion

In all cases, the hydrogen permeance was higher than that of nitrogen or CO_2_, having a ratio similar to that predicted by the Knudsen flow, i.e., the square root of the molecular weight ratio. On the contrary, the water permeance was similar to the hydrogen permeance in the case of the single-layer membrane and even higher in the case of the double-layer membrane, in spite of the much smaller molecular weight of hydrogen. This result implies that an additional mechanism for water permeation occurred, which was probably a solution–diffusion mechanism in the case of water permeation. The double-layer membrane had much lower permeance for permanent gases (typically one order of magnitude smaller), while the water permeance was only decreased by a factor of two in some cases; therefore, much higher H_2_O/H_2_ and H_2_O/CO_2_ selectivity was achieved with the double-layer membrane. This suggests that double-layer membranes have fewer defects. 

**Figure 4:**
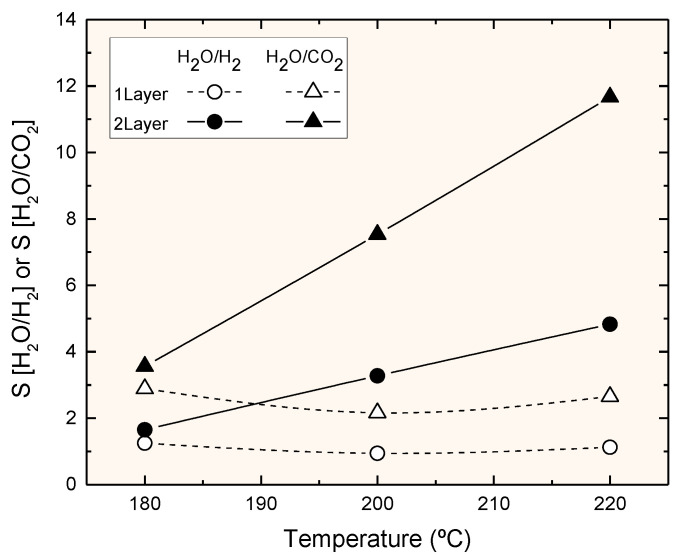
Selectivity (H_2_O/H_2_ or H_2_O/CO_2_) for the two membranes as a function of temperature.

**Figure 5:**
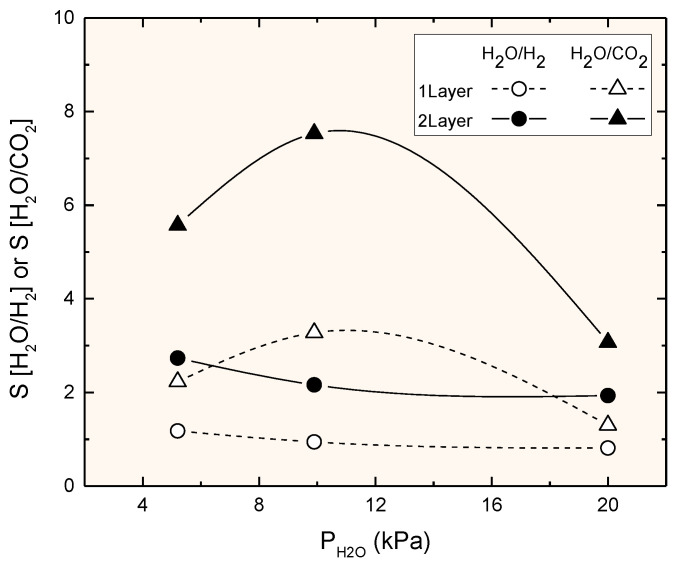
Selectivity (H_2_O/H_2_ or H_2_O/CO_2_) for the two membranes as a function of water partial pressure.

The above results become clearer by looking to the H_2_O/H_2_ and H_2_O/CO_2_ selectivity levels, as reported in Figure 4 and Figure 5. Both selectivity levels increased with temperature in the studied interval, which is an interesting result for this kind of membrane. In particular, it seems that this kind of membrane would resist higher temperatures than the Nafion membrane employed in a previous study [12], allowing its use at temperatures more suitable for achieving reasonable reaction rates with conventional catalysts. The H_2_O/CO_2_ selectivity was between 2 and 2.5 times higher than the H_2_O/H_2_ selectivity, in agreement with the hypothesis that a large part of both CO_2_ and H_2_ permeated through small pores, corresponding to the Knudsen flow; however, since that ratio was smaller than 4.6 (the square root of the molecular weight ratio), a contribution from the convective flow may have occurred. 

The effects of the partial water pressure were different in both membranes. In the single-layer membrane, an increase in the water partial pressure caused a decrease in water permeance, i.e., the flux of the water did not increase linearly with the partial water pressure, suggesting that water solubility has a maximum value, as is the case for Langmuir adsorption isotherms. On the contrary, with the double-layer membrane, the maximum permeance level was achieved at an intermediate partial water pressure. 

The effects of temperature on the permeance of permanent gases were small. For the single-layer membrane, the nitrogen permeance decreased slightly with temperature, as may be expected for Knudsen flow (Equation (1)), while the hydrogen permeance slightly increased, suggesting that hydrogen permeation was assisted by activated flow, i.e., through pores with a size similar to that of the permeating molecule. For the double-layer membrane hydrogen permeance decreased with temperature, suggesting that the diffusion through micropores was less significant than for the single-layer membrane. 

The comparison of these membranes with previous results with zeolite membranes (18) showed that the selectivity was a bit smaller. As an example, the H_2_O/H_2_ selectivity with a zeolite membrane at 220 °C was ca. 20 [18], while the result obtained with the double-layer silicone membrane in this work was only 5; however, the water flux (2.5 mmol/m^2^ s) was similar to that obtained with the zeolite membrane. The ability to achieve such flux values is interesting, since the silicone–ceramic composite membrane can still be optimized, meaning it could be competitive with the zeolite membrane.

## 5. Conclusions

Silicone rubber membranes deposited on ceramic supports can selectively remove water from a mixture of gases containing hydrogen and CO_2_ at temperatures up to 220 °C; therefore, they may be useful for membrane reactors for methanol synthesis by CO_2_ hydrogenation. The best obtained selectivity for H_2_O/H_2_ was 4.8 at 220 °C, with a water permeance level of 1.40·10^−7^ mol/m^2^ s Pa. Nitrogen permeation suggests the existence of small pores (defects) that decrease the selectivity of H_2_O/H_2_. Further optimization of the preparation procedure is needed to achieve a membrane with comparable selectivity to that obtained with zeolite membranes. If such polymer–ceramic membranes are achieved, they could have many applications in membrane reactors for processes where the removal of water improves the achievable yield [23,24,25]. 

## Figures and Tables

**Table 1:** Permeance (J) of each gas obtained with the single-layer silicone membrane with different temperatures and water partial pressures in the feed.

T (°C)	P_H2O_ (kPa)	J (10^−7^ mol/m^2^ s Pa)			
		H_2_	N_2_	CO_2_	H_2_O
180	9.9	3.10	1.02	1.34	3.87
200	9.9	3.37	0.93	1.47	3.18
220	9.9	3.40	0.96	1.44	3.82
200	5.2	3.32	0.87	1.43	3.91
200	20	3.24	0.75	1.36	2.63

**Table 2:** Permeance (J) of each gas obtained with the double-layer silicone membrane with different temperatures and water partial pressures in the feed.

T (°C)	P_H2O_ (kPa)	J (10^−7^ mol/m^2^ s Pa)			
		H_2_	N_2_	CO_2_	H_2_O
180	9.9	0.35	0.17	0.16	0.57
200	9.9	0.35	0.16	0.15	1.13
220	9.9	0.29	0.13	0.12	1.40
200	5.2	0.35	0.17	0.14	0.78
200	20	0.33	0.17	0.14	0.43

## Data Availability

Data is contained within the article.
